# Totally 3-dimensional endoscopic aortic valve replacement for situs inversus totalis using a 2-window port configuration

**DOI:** 10.1016/j.xjtc.2025.09.019

**Published:** 2025-10-03

**Authors:** Akitoshi Yamada, Chihiro Okubo, Ryo Tohma, Hidekazu Nakai, Yoshihisa Morimoto, Kunio Gan, Tatsuro Asada

**Affiliations:** Department of Cardiovascular Surgery, Kita-Harima Medical Center, Hyogo, Japan

**Figure:**
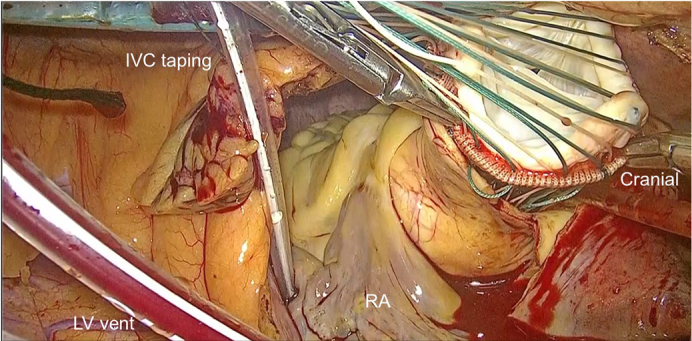
Prosthetic valve implanted in the normal position despite mirror-image anatomy of SIT.

**Central Message**

Totally endoscopic AVR is feasible in situs inversus totalis with careful planning and strategic technical modifications.

Situs inversus totalis (SIT) is a rare congenital anomaly with mirror-image organ arrangement (∼1 in 6500-25 000).^1^ This complicates minimally invasive cardiac surgery (MICS), where port planning and ergonomic maneuverability are critical. Totally endoscopic aortic valve replacement (AVR) provides magnified vision but faces technical challenges like blind spots and restricted instrument mobility. Herein, we report the first case totally endoscopic MICS AVR in a patient with SIT, highlighting preoperative simulation, mirror-adapted port configuration, and intraoperative strategies, including unidirectional suture flow and controlled suture sequencing.

## Case

**Figure 1:**
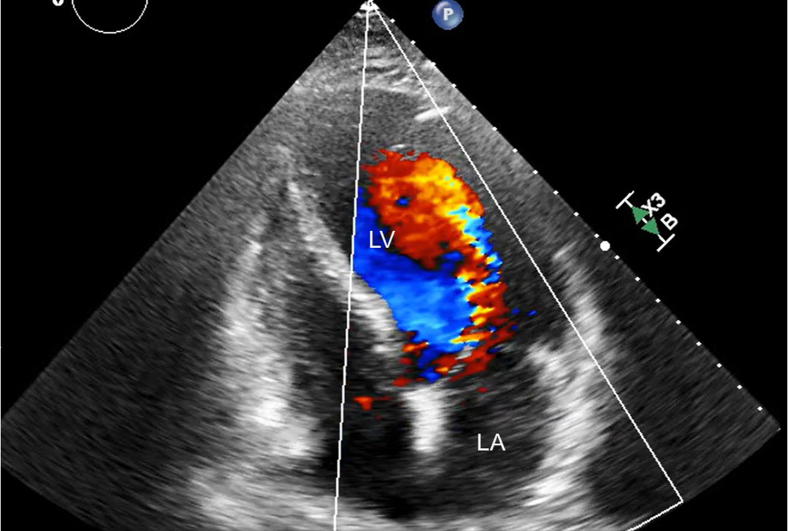
Transthoracic echocardiographic image showing severe aortic regurgitation. *LV*, Left ventricle; *LA*, left atrium.

**Figure 2:**
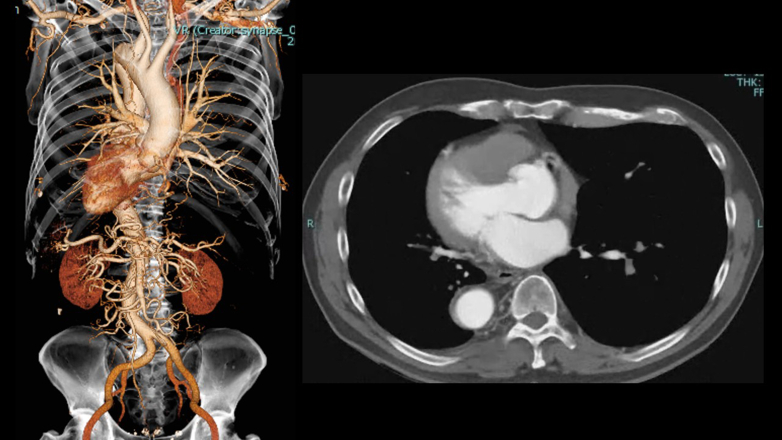
Situs inversus totalis confirmed by 3-dimensional reconstruction and axial computed tomography images showing mirror-image positioning of thoracoabdominal organs.

**Figure 3:**
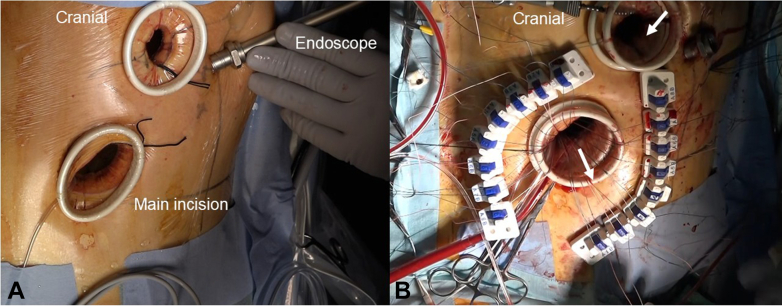
A, Surgical ports were arranged to allow a 2-window access from the left side. B, The suture pathway for valve fixation is indicated by *white arrows*: the needle is inserted through the right-side window, anchored to the aortic annulus, and then extracted through the main incision on the left.

A 69-year-old man (166 cm, 73 kg, body mass index 26.4) with no significant comorbidities presented with exertional dyspnea. Transthoracic echocardiography revealed severe aortic regurgitation with an effective regurgitant orifice area of 0.48 cm^2^ and a regurgitant fraction of 51% (Figure 1). The left ventricle was dilated (left ventricular diastolic dysfunction 57.1/42.7 mm), and the ejection fraction was reduced to 46%. Prolapse of the right coronary cusp was suspected. Chest and abdominal computed tomography confirmed SIT (Figure 2). Preoperative simulation confirmed the feasibility of adapting our standard 3-port totally endoscopic AVR technique^2^ to the mirror-image anatomy. To accommodate the right-handed surgeon's natural ergonomics, a 2-window approach^3^ was adopted, consisting of a 4-cm working/camera port in the left fourth intercostal space and a 2-cm auxiliary port in the left third intercostal space (Figure 3, *A*). The procedure was performed entirely under totally 3-dimensional endoscopic vision. Cardiopulmonary bypass was established via left femoral arterial and venous cannulation. Following aortic crossclamping, antegrade selective cardioplegia was administered, and the patient was cooled to 30 °C. After excising the native aortic valve, a 25-mm Epic bioprosthetic valve (Abbott Vascular) was implanted using 15 2-0 Ethibond (Ethicon) mattress sutures with polytetrafluoroethylene pledgets. The sutures were secured with Cor-Knot fasteners (LSI Solutions). All needles were introduced via the third intercostal port and retrieved through the fourth intercostal port to establish a unidirectional suture flow (Figure 3, *B*). Sutures were placed in the sequence of the right coronary cusp, noncoronary cusp, and left coronary cusp to enhance intraoperative organization and prevent suture entanglement (Video 1). The total operative time was 246 minutes, with cardiopulmonary bypass and aortic crossclamp times of 194 and 141 minutes, respectively. Echocardiography showed neither paravalvular leakage nor transvalvular regurgitation, and the patient was transferred to the general ward on postoperative day 1 and discharged on postoperative day 6. This report was approved by the institutional review board of the Kita-Harima Medical Center (07-21) (approved August 8, 2025). Informed consent was obtained from the patient and his family for the publication of this case report.

## Discussion

Mirror-image anatomy in SIT complicates MICS by reversing landmarks and spatial orientation. In totally endoscopic AVR, limited space and blind spots further amplify these difficulties, in contrast to direct-vision or video-assisted approaches. Thus, a steep learning curve is required for totally endoscopic MICS AVR. Although video-assisted AVR^4^ and totally endoscopic mitral surgery^5^ have been described in patients with SIT, to the best of our knowledge, this is the first report of a successful totally endoscopic AVR in such a case.

Several technical strategies facilitated the safe and efficient conduct of this procedure. First, we employed a 2-window port configuration that maintained intuitive instrument movement for a right-handed surgeon while minimizing interference, despite the reversed thoracic anatomy. Second, unidirectional suture management was implemented by separating needle insertion and retrieval ports. Along with a deliberate suture sequence involving the right coronary cusp, followed by the noncoronary cusp and then the left coronary cusp, this strategy minimized suture entanglement and improved field organization. Third, the use of Cor-Knot fasteners enabled secure and rapid suture fixation, which streamlined the procedure and preserved clarity in the operative field. Furthermore, the rib-sparing, totally endoscopic approach likely contributed to reduced postoperative pain, superior cosmetic outcomes, and early ambulation. Anticipating prolonged ischemic time due to the procedural complexity, we applied moderate systemic hypothermia and antegrade cardioplegia to ensure adequate myocardial protection.

To safeguard against potential technical difficulties associated with the totally endoscopic approach, a predefined bailout strategy was established. If intraoperative challenges impeded safe completion of the procedure, the 2 port sites were designed to be convertible into a single working incision, allowing for anterior disarticulation of the fourth rib and transition to a direct-vision MICS approach. This contingency plan provided a practical safety margin while preserving the minimally invasive nature of the procedure without necessitating full sternotomy.

The patient's uneventful recovery highlights the safety of this approach. This case shows that, with careful planning and technique, totally endoscopic AVR can be performed safely and reproducibly, demonstrating its applicability even in SIT.

## Conclusions

Totally endoscopic AVR in a patient with SIT—despite the inherent challenges of reversed anatomy—can be performed safely and efficiently by combining technical strategies such as careful preoperative simulation, strategic port placement, unidirectional suture flow, and optimized suture sequencing. This first reported case of its kind highlights the technical ingenuity and operative strategies underlying this approach and suggests that such innovations may expand the applicability of MICS to patients with complex anatomy.

## Conflict of Interest Statement

The authors reported no conflicts of interest.

The *Journal* policy requires editors and reviewers to disclose conflicts of interest and to decline handling or reviewing manuscripts for which they may have a conflict of interest. The editors and reviewers of this article have no conflicts of interest.

## Supplementary Data

Video 1 Intraoperative video demonstrating key steps of totally endoscopic aortic valve replacement in a patient with situs inversus totalis, including valve excision and prosthesis implantation. Video available at: https://www.jtcvs.org/article/S2666-2507(25)00439-0/fulltext.
