# Supplementary figures and images for: Totally 3-dimensional endoscopic aortic valve replacement for situs inversus totalis using a 2-window port configuration
Source: JTCVS Tech. 2025 Oct 3;34:81–3. doi: 10.1016/j.xjtc.2025.09.019 (PMC12683039; doi:10.1016/j.xjtc.2025.09.019)

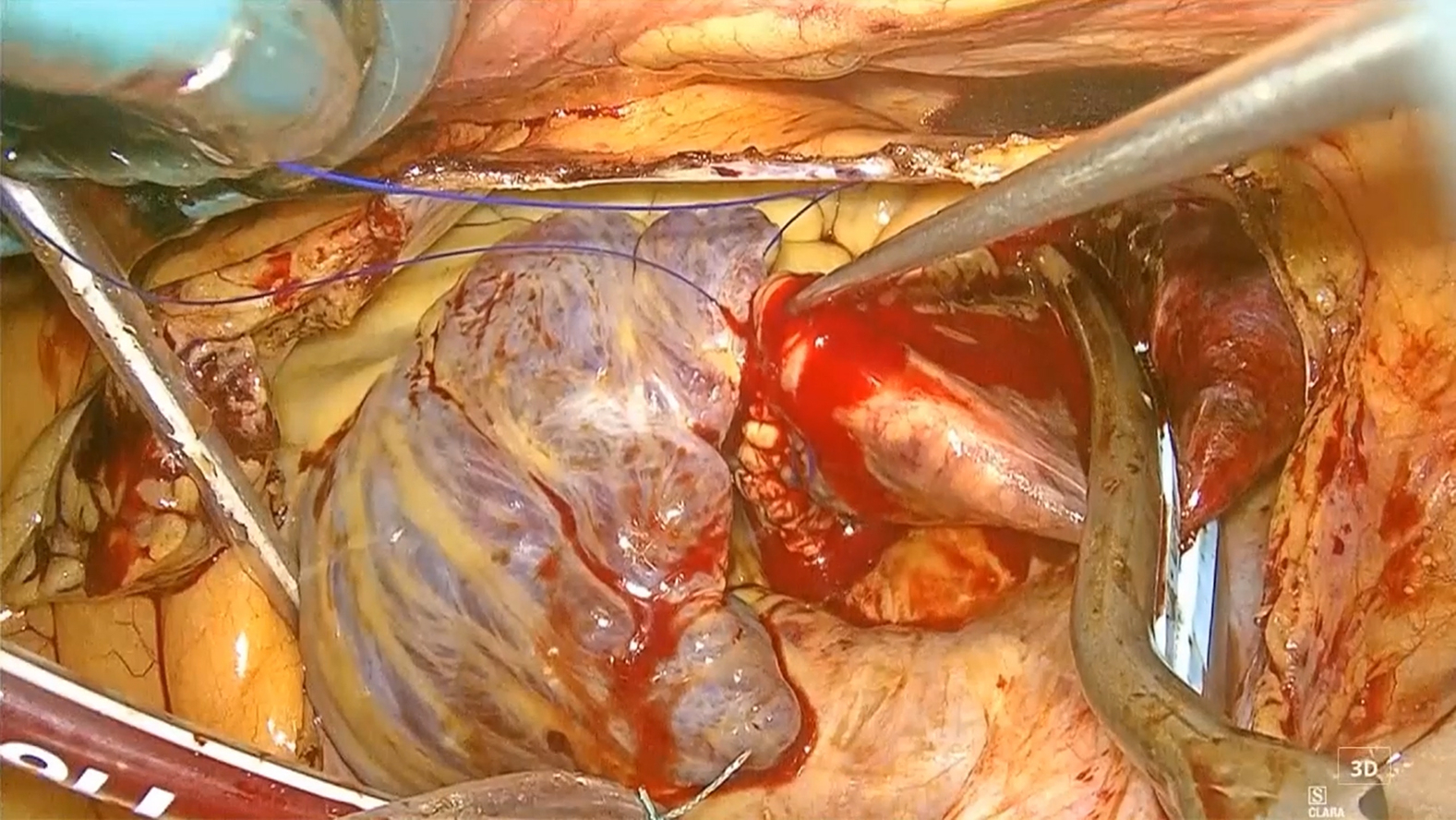

Supplement: Video 1 — Intraoperative video demonstrating key steps of totally endoscopic aortic valve replacement in a patient with situs inversus totalis, including valve excision and prosthesis implantation. Video available at: https://www.jtcvs.org/article/S2666-2507(25)00439-0/fulltext. [file fx2.jpg]
